# Aeroallergen sensitivity patterns in Gulf countries: A systematic review

**DOI:** 10.1002/clt2.70033

**Published:** 2025-02-20

**Authors:** Tahira Khurram, Ghalia Missous, Nicholas van Panhuys, Mohammed Yousuf Karim

**Affiliations:** ^1^ Primary Health Care Corporation Doha Qatar; ^2^ Laboratory of Immunoregulation Sidra Medicine Doha Qatar; ^3^ Hematology, Immunology and Transfusion Division Sidra Medicine Doha Qatar

**Keywords:** aeroallergens, aerobiology, allergic rhinitis, asthma, GCC countries, serum IgE, skin prick test

## Abstract

**Background:**

Successful management of allergic diseases necessitates accurate diagnosis, implementation of appropriate allergen avoidance techniques, and medical therapies. However, data availability regarding aeroallergens in the Gulf Cooperation Council (GCC) countries is limited.

**Methods:**

We conducted a systematic review of studies conducted in Gulf countries on individuals diagnosed with or tested for aeroallergen sensitivities, focusing on prevalence and respiratory health impacts. The search strategy followed the PRISMA guidelines and was conducted across PubMed, Scopus, Web of Science, and Google Scholar from inception to November 12, 2023.

**Results:**

A total of 27 studies, both adult and pediatric, were included in this systematic review. Aeroallergen sensitization was assessed using skin prick testing (SPT) in 15 studies; 5 used in vitro methods, 2 employed both, 4 relied on self‐reports, and 1 on aerobiological monitoring. Sensitization rates varied considerably, influenced by factors such as age, demographics, and location. Sensitization was noted to allergens shared with Western populations, and to those native to the region for example, house dust mite sensitization ranged from 15% to 78%, Salsola from 13% to 78%. Up to 65.1% of allergen‐positive individuals demonstrated polysensitization. Sensitization patterns differed between indigenous populations and expatriates, with local allergens being more prevalent among natives. Sensitization rates were lower in younger children but increased with age.

**Conclusion:**

Our systematic review highlights the crucial importance of providing allergen‐sensitivity information that is specifically tailored to the local environment. This tailored approach can improve clinical diagnosis, enable appropriate allergen avoidance and immunotherapy strategies, and result in potential cost savings.

## 1 INTRODUCTION

Numerous substances of diverse origins continuously circulate in the atmosphere, forming atmospheric aerosols.^1^ Airborne allergens are a crucial element of aerosols. These allergens are biologically derived substances, such as pollen, mold spores, dust mite feces, and animal dander. When these allergens enter the human body through inhalation, they can stimulate the immune response in genetically predisposed individuals, leading to the development or exacerbation of allergic diseases, such as allergic rhinitis and asthma.^1^, ^2^ The prevalence and concentration of these allergens in the atmosphere are influenced by various environmental factors.

The allergenic content of the atmosphere varies significantly based on climate, geography, and vegetation. For instance, regions with dense vegetation may have higher concentrations of pollen, while humid environments may facilitate the growth and dispersion of mold spores. The composition of biological particles in the atmosphere exhibits both spatial and temporal variability, meaning that allergen levels can differ widely between locations and change over time. The desert climate in the Gulf Cooperation Council (GCC) states, together with increasing urbanization, has substantial implications for potential variation in aeroallergen levels and consequent allergic responses.^3^, ^4^

Several primary factors influence the behavior and concentration of airborne allergens. Seasonal changes play a critical role, as plant phenology determines when pollen is released into the atmosphere. Surrounding vegetation types and density also affect the types and amounts of allergens present. Additionally, meteorological conditions, such as temperature, humidity, wind patterns, and precipitation, can impact the dispersion and deposition of allergens.

Given the health implications of airborne allergens, monitoring their presence in the atmosphere has become essential. Pollen monitoring, in particular, has become a standard practice in many countries enabling the collection of data that helps predict allergen levels and inform public health strategies.^5^ Allergic diseases, including conditions such as allergic rhinitis, asthma, and atopic dermatitis, significantly impact the quality of life of affected individuals. Allergic inflammation and reactions to allergen exposure can manifest within the target organ, such as in allergic rhinitis and allergic asthma, or systemically, as observed in anaphylaxis.^6^ Avoiding specific allergens can be an effective strategy for preventing allergies and managing symptoms. Reports indicate that avoiding significant allergens can lead to clinical remission and, in many instances, decrease bronchial hyper‐reactivity.^7^

Successful management of allergic diseases in the Gulf region necessitates accurate diagnosis, implementation of appropriate allergen avoidance techniques, and medical therapies.^8^ However, data availability regarding aeroallergens in the GCC countries is limited. This systematic review aims to assess the prevalence of aeroallergen sensitization in the GCC states, providing a foundation for better diagnosis and management of allergic diseases in this region.

## 2 METHODS

### 2.1 Search strategy and eligibility criteria

The study protocol was officially registered in the Prospective Register of Systematic Reviews (PROSPERO) under the registration ID CRD42024512116. A systematic search strategy, following the Preferred Reporting Items for Systematic Reviews and Meta‐Analyses (PRISMA) guidelines^9^ was employed across PubMed, Scopus, Web of Science, and Google Scholar from inception until 12 November 2023. Boolean operators and Medical Subject Headings (MeSH) terms were utilized to enhance search precision. The search terms included “Aeroallergen,” “Airborne allergen,” “Allergen sensitivity,” “Gulf countries,” “Middle East countries,” “Saudi Arabia,” “United Arab Emirates,” “Qatar,” “Bahrain,” “Kuwait,” and “Oman.” The review specifically focused on peer‐reviewed studies conducted in these regions, encompassing individuals of all ages diagnosed with aeroallergen‐related conditions or tested for sensitivities. The investigation explored patterns of aeroallergen sensitivity and their impact on respiratory health, with outcomes encompassing prevalence rates and associated health impacts. Exclusions included non‐peer‐reviewed studies, reviews, conference abstracts, letters, editorials, opinion pieces, and studies involving populations outside Gulf countries or conducted on animals. Additional relevant references were identified based on suggestions from co‐author MYK.

### 2.2 Data collection

Two investigators (TK and GM), independently screened titles and abstracts, evaluated full texts of potentially eligible records, and extracted data using an extraction table established by consensus among all authors. This table included relevant datasets, such as author and publication year, country, study design, participant characteristics, and aeroallergens tested. Any disagreements were resolved through consensus among the authors.

### 2.3 Quality assessment and risk of bias

The methodological quality and risk of bias were assessed by TK, GM, and NVP using checklists adapted from the Joanna Briggs Institute (JBI) critical appraisal tools for cross‐sectional and case‐control studies.^10^, ^11^ Since no specific checklist exists for chart reviews, we applied the checklist for analytical cross‐sectional studies to evaluate two such studies.^12^, ^13^ Although we acknowledge the differences in design and methodology between chart reviews and cross‐sectional studies, this checklist offers a structured framework for quality appraisal and bias assessment. We also recognize the limitations of this approach, particularly the potential mismatch between some criteria and the nature of chart reviews.

Checklist responses were categorized as ‘yes,’ ‘no,’ or ‘unclear.’ Study quality was evaluated based on design relevance, reporting, and risk of bias, with methodological quality categorized as high, medium, or low depending on the criteria met and the presence of bias or design flaws. Final decisions on study inclusion in the systematic review were based on both the quality appraisal results and the authors' collective judgment of each study's significance and methodological rigor.

## 3 RESULTS

### 3.1 Search results and study characteristics

**FIGURE 1:**
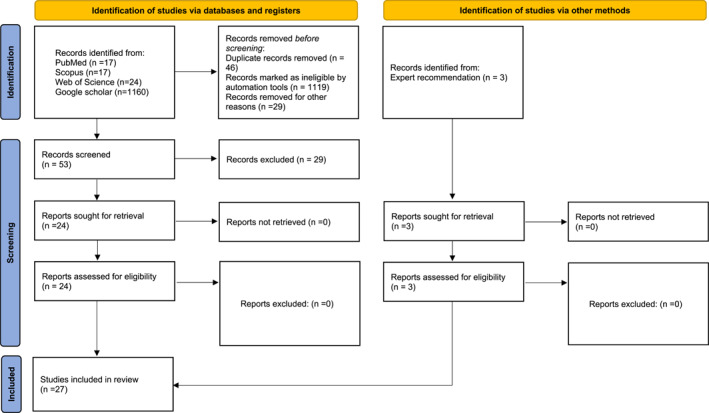
PRISMA Flow Diagram illustrating the study selection process.

The database search identified 1218 articles, of which 46 were duplicates, and 1119 were ineligible based on the exclusion criteria. An additional 29 records were removed for other reasons, including studies outside the GCC, those not focused on aeroallergen sensitization, and misclassified records due to author names resembling country names. A total of 53 publications were thoroughly screened, with three more based on MYK's recommendation. Figure 1 presents a flow diagram of the systematic study selection process following the PRISMA 2020 guidelines.^9^ Twenty‐seven publications were included in the final review. Twenty‐four studies utilized a cross‐sectional design, while one study employed a case‐control design, and two studies were retrospective chart reviews (Table 1).

**TABLE 1:** Study characteristics and key findings.

Author name publication year	Country	Study design	Target population/disease	Sample size	Age (years)	Sex (F/M)	Methods used for aeroallergen identification	Disease prevalence/Summary statistics	Aim of study	Key findings
Al‐Dowaisan et al. 2004^14^	Kuwait	Cross‐sectional	Asthma Allergic rhinitis	451	29.5	192/259	SPTs	Isolated AR = 44.34%, AR and asthma = 47.01%, Isolated asthma = 8.64%	To assess SPTs results for allergens relevant to Kuwait's vegetation surveys and aerobiological studies.	‐ Indoor allergens play minor role in respiratory allergies in Kuwait. ‐ Allergic patients are sensitized to pollens. ‐ Salsola pollen elicits the strongest and most frequent reactions. ‐ Peak symptoms of seasonal AR occur in autumn.
Al‐Frayh et al. 1992^15^	KSA	Cross‐sectional	Asthmatic children	240	6–13 years	NS	SPTs	Sensitization to at least one allergenic extract: 59.2% from central region (Riyadh) 72.5% from Western region (Makkah)	To compare the immediate skin test reactivity to inhalant allergens among asthmatic children in two different regions of KSA.	‐ House dust mites were a prominent allergen in the Western region, less so in the central region. ‐ Cat fur sensitivity was notable across both regions. ‐ Reactions to cockroach allergens and cotton flock occurred in both regions.
Al‐Frayh et al. 1999^16^	KSA	Cross‐sectional	Allergic patients with bronchial asthma	473	1 to >18 years	167/253	‐ SPTs ‐ RAST ‐ Air sampling	‐ SPT results: Positive reactions to Prosopis juliflora were 76.1% in Qassim, 37.5% in Gizan, 29.1% in Abha, and 11.1% in Hofuf. ‐ RAST results: 11.3% of patients showed positive results, with varying levels of specific IgE antibodies.	To evaluate the sensitization to Prosopis juliflora pollen among allergic patients in different regions of Saudi Arabia.	‐ High sensitization rates to Prosopis juliflora pollen were observed, particularly in Qassim (76.1%). ‐ Multiple sensitizations to other pollen allergens, such as Chenopodium album and Cynodon dactylon, were also noted. ‐ Airborne Prosopis pollen concentrations peaked in April, exceeding 90 grains/m³ in Gizan. ‐ The study confirmed Prosopis juliflora as a significant allergen in Saudi Arabia.
Al‐Ghamdi et al. 2022^17^	KSA	Cross‐ sectional (PB)	Adults with CR	969	37.63 ± 12.26	251/718	‐ Aeroallergen‐specific IgE immunoassay. ‐ Total IgE and cytokine ELISA. ‐ Eosinophilic count.	CR = 35.8%	‐ To determine the prevalence of CR among adults in southwestern Saudi Arabia. ‐ To identify specific aeroallergen sensitizations associated with CR.	‐ There is a significant association between CR and sensitization to outdoor aeroallergens, including mesquite, Chenopodium, Ragweed, Pigweed, Russian thistle, Bermuda grass, Timothy grass, and Rye. ‐ Indoor aeroallergens were not significantly associated with CR. ‐ Higher total IgE levels and eosinophil counts were observed in adults with CR.
AlKhater, 2017^18^	KSA	Cross‐sectional	Asthmatic children	100	8.98 ± 2.93	32/68	SPTs	Sensitization to at least one allergenic extract 86%	To determine the prevalence of sensitization to common aeroallergens among asthmatic children.	‐ House dust mites were the most common allergens. ‐ Indoor allergens were more common in younger children.
Almehizia et al. 2019^19^	KSA	Cross‐sectional	Allergic rhinitis	2849	29.22 ± 10.1	1691/1158	Self‐report	Intermittent AR = 54%, Persistent AR = 46%	To explore the disease characteristics of AR within saudi community. To identify non‐conventional coping measures used by individuals to alleviate AR symptoms.	‐ Dust was the leading trigger, followed by pollen, mold, and fur. ‐ Conventional coping measures include antihistamines (55.1%) and clinic visits (1.9%) ‐ Unconventional measures include shower/humidification (12.9%) and herbal hot drinks (9.3%). ‐ Older and overweight participants reported more persistent AR. ‐ Females reported milder forms compared to males.
Almogren, 2009^20^	KSA	Cross‐sectional	Asthma and allergic rhinitis	139	27 ± 12	78/61	SPTs	Vb sensitization to at least one allergenic extract 75%	To determine the pattern of SPTs reactivity to aeroallergens in patients with asthma and rhinitis in Riyadh.	‐ House dust mites were the most common indoor allergen. ‐ Prosopis juliflora and Bermuda grass are most common outdoor allergens.
Al‐Nesf et al. 2020^21^	Qatar	Cross‐sectional	Adults with asthma and allergies	940	30.0 (20–61)	510/430	‐ Airborne pollen monitoring ‐ SPTs	21.7% showed an allergic sensitization to pollen.	To assess the prevalence and types of airborne pollen in Qatar and correlate these with sensitization in patients presenting with respiratory allergies.	‐ Amaranthaceae and Poaceae were the most prevalent pollen types detected. ‐ Higher sensitization rates to these pollen types, with a notable impact on asthma and AR cases.
Al‐Nesf et al. 2022^22^	Qatar	Cross‐sectional (PB)	Middle school children	644	12–15 years	NS	‐ Aerobiological monitoring ‐ Modified ISAAC questionnaire	‐ 331 students reported positive symptoms: AR = 62.8%, lifetime wheeze = 28.1%, Eczema = 26.6%. ‐ Asthma prevalence was significantly higher in Qatari children (39.8%) compared to non‐Qatari children (26.7%).	To investigate the correlation between the seasonality of allergic symptoms and the levels of airborne pollen and fungal spores among middle schoolchildren in Qatar.	‐ AR symptoms were strongly linked to high airborne fungal spores in November. ‐ Outdoor aeroallergens and environmental factors contribute significantly to children's allergic symptoms.
Alqahtani, 2016^23^	KSA	Cross‐sectional (PB)	School children	1700	12.2 ± 3.3	849/851	SPTs	Asthma = 27.5%, AR = 6.3%, AD = 12.5%	To study the prevalence and risk factors of allergic diseases in saudi schoolchildren in Najran, and their allergen sensitization.	‐ Grass pollens, cat fur, and house dust mites are the most frequent allergens. ‐ Sensitization to grass pollens is especially high in Najran due to its agricultural landscape and high altitude. ‐ Allergic diseases are linked to various risk factors, including male sex, fast food consumption, presence of pets, and exposure to vehicle emissions.
AlShatti and Ziyab, 2020^24^	Kuwait	Cross‐sectional (PB)	School children	3864	12.0 (11.0, 14.0)	2169/1695	ISAAC questionnaire	Doctor diagnosed Asthma = 23.5%, Rhinitis = 24.9%, Eczema = 19.5%	To evaluate the relationship between pet‐keeping and allergic disease symptoms in adolescents	‐ Cat ownership linked to increased wheezing, rhinitis symptoms, and eczema diagnoses. ‐ Rabbit and bird ownership associated with higher rates of asthma symptoms, severe eczema, and rhinitis.
Al‐Saleh et al. 2019^25^	Bahrain	Case control	Adults with pulmonary and chest diseases	232	18–92 years	108/44	Phadia 250 fluoro‐enzyme immunoassay	‐ 16% of asthmatic patients were sensitized to Aspergillus fumigatus. ‐ 10.1% had allergic bronchopulmonary aspergillosis (ABPA). ‐ 75.6% were sensitized to pollen grains, and 22.3% had food allergies.	To assess the prevalence of Aspergillus sensitization and ABPA in asthmatic patients in Bahrain.	‐ The study highlighted the significant presence of Aspergillus fumigatus sensitization among asthmatic patients and the prevalence of ABPA
Al‐Tamemi et al. 2008^26^	Oman	Cross‐sectional	All age groups with asthma, AR and rhinoconjunctivitis	689	Mean 30.0	375/314	SPTs	Asthma = 39.2%, AR = 61.3%, Rhinoconjuctivitis = 1.9%	To identify prevalent inhalant allergens and assess their sensitization patterns in Omani patients	‐ High rates of sensitization to house dust mites and various other inhalant allergens were found. ‐ The pattern of sensitization in Oman is consistent with other regions in the Arabian Peninsula.
AlMehdi et al. 2005^27^	UAE	Cross‐sectional	Airborne allergies	477	NS	NS	RAST and total IgE Air sampling	‐ Date‐Palm pollen concentration was approximately 800 counts/m³ within farms, dropping by 80% at 100 m and almost absent beyond 200 m from the farm. ‐ 2.3% of patients tested positive for Date‐Palm pollen, 16.1% for grass pollens, and 12.4% for mesquite tree pollens	To investigate the allergenicity of Date‐Palm pollen in the UAE and its impact on airborne allergic patients.	‐ Date‐Palm pollen showed low allergenic potential with only 2.3% of tested patients showing positive RAST results. ‐ The pollen concentration significantly decreases beyond the immediate vicinity of Date‐Palm farms. ‐ The smooth texture and heavy mass of Date‐Palm pollen grains likely contribute to their limited dispersal and low allergenic impact.
Hasan and Rizwan, 2015^12^	Bahrain	Retrospective chart review	All age groups with AR	600	10–50 years	312/288	SPTs	Bronchial asthma = 31%, Eczema = 9%, Asthma and eczema = 3%	To evaluate the incidence of common food and inhalant allergies in patients with resistant allergic rhinitis.	‐ 54% of patients were allergic to more than one inhalant allergen: 33% were allergic to HDM, and 21% were allergic to Salsola kali. ‐ Food allergies were less common, with walnut being the most prevalent food allergen (8%).
Hasnain et al. 1994^28^	KSA	Cross‐sectional	Asthmatic children	240	6–13 years	NS	SPTs Air sampling	‐ Cladosporium prevalence: 25% in Riyadh, 37.5% in Jeddah, 41.2% in Al‐Khobar. ‐ Skin Test reactivity: 5.8% in Riyadh, 31.3% in Makkah.children in Riyadh and Makkah had 5.8% and 31.3% Cladosporium extract skin reactivities.	To investigate the prevalence and seasonal variation of Cladosporium spores in Saudi Arabia and assess their potential role as airborne allergens.	‐ Cladosporium spores accounted for 37.5% of airborne spores in Jeddah and 41.2% in Al‐Khobar, with significant seasonal peaks. ‐ Positive skin test reactions to Cladosporium correlated with higher humidity and spore concentrations. ‐ Cladosporium was identified as a significant aeroallergen.
Hasnain et al. 1998^29^	KSA	Cross‐sectional	Allergic diseases, including bronchial asthma and AR	616	1 to >18 years	249/367	SPTs Air sampling	‐ Alternaria spores: 1.9%–9.6% of total spores, peaking at over 500 spores/m³ in Jeddah. ‐ SPT reactions: 19.16% mild, 1.79% moderate, and 0.65% strong.	To investigate the prevalence and sensitization potential of airborne Alternaria spores in KSA.	‐ Concentrations of Alternaria spores varied, peaking at 9.6% in April at KFSH&RC. ‐ 21.6% of patients showed positive skin test reactions. ‐ Alternaria spores are a significant allergen, linked to asthma and allergic rhinitis in the region.
Hasnain et al. 2004^30^	KSA	Cross‐sectional	Allergic individuals	605	NS	243/362	SPTs Air sampling	‐ Cladosporium prevalence accounted for 20%–41.2% of total fungal spores, varying by location. ‐ 19.67% of patients showed positive reactions, with 16.67% mild, 2.00% moderate, and 1.00% strong reactions.	To evaluate the allergenicity of Cladosporium spores in Saudi Arabia and their impact on respiratory allergies.	‐ Cladosporium was the most prevalent fungal spore in the air, particularly in coastal cities like Jeddah and Al‐Khobar. ‐ The maximum concentration of Cladosporium spores reached 14,000 spores/m³. ‐ Sensitization to Cladosporium was identified in 19.67% of the tested population, with varying degrees of severity. ‐ The study highlights the need for detailed allergen‐specific diagnostic methods and the potential for Cladosporium to cause allergic reactions.
Hasnain et al. 2012^4^	KSA, UAE and Sudan	Cross‐sectional	Patients with allergic respiratory diseases	492	14–49 years	276/256	SPTs Air sampling	‐ *Pollen Sensitization*: Chenopodium murale: 32% in Khartoum, Sudan Salsola imbricata: 30% in Riyadh, Saudi Arabia Prosopis juliflora: 24% in Riyadh, 19% in Dammam, Saudi Arabia ‐ *Mold Sensitization*: Cladosporium spp.: 42% in Khartoum, Sudan Aspergillus fumigatus: 40% in Khartoum Alternaria alternata: 38% in Khartoum	To evaluate the prevalence of sensitization to indigenous pollen and molds, as well as other indoor and outdoor allergens in patients with allergic respiratory diseases across different regions.	‐ High sensitization rates were observed for both indigenous pollen and molds, particularly in Khartoum for molds. ‐ The study showed that Salsola imbricata and Chenopodium murale were significant pollen allergens in Saudi Arabia and Sudan, respectively. ‐ High sensitization to HDM was also noted, especially in humid areas like Khartoum and Jeddah.
Kerkadi et al. 2009^31^	Qatar	Cross‐sectional	Children with asthma and allergies	134	1 month to 10 years	95/39	RIDA Allergy Screen‐test	Asthma = 56.7% Eczema = 14.4%, Wheezing = 61.9%	To estimate the prevalence of sensitization to common food and inhalant allergens among children in Qatar.	‐ Common food allergens: Cheese, milk, casein, peanut, nut, fish, banana, egg yolk. ‐ Prevalent inhalant allergens: pets (cats, dogs), dust. ‐ Food allergen sensitization decreases with age; inhalant allergen sensitization increases with age. ‐ Significant link found between food and inhalant allergen sensitization, especially in children with a family history of allergy.
Koshak, 2006^32^	KSA	Cross‐sectional	Asthmatic adults	171	12–64 years	110/61	SPTs Total serum IgE Phadiatop: Specific multi‐allergen IgE test	‐ 86% of patients had positive reactions, indicating atopic asthma. ‐ Elevated IgE levels in 60% of cases, with significantly higher mean IgE levels in AA (722 ± 888 IU/mL) compared to non‐atopic asthma (NAA) (43 ± 49 IU/mL). ‐ Phadiatop results were positive in 88% of AA cases, with 88% sensitivity and 87.5% specificity.	To explore the role of in vitro IgE tests in identifying atopic asthma (AA) and differentiating it from non‐atopic asthma (NAA).	‐ High prevalence of atopic asthma was identified, with significant IgE sensitization to inhalant allergens. ‐ In vitro tests like Phadiatop demonstrated high sensitivity and specificity, making them useful for identifying allergen sensitization, especially when SPT is unavailable. ‐ Elevated IgE levels correlated with asthma severity, supporting their use in clinical assessment and management of asthma.
Mahboub et al. 2014^33^	UAE	Cross‐sectional	AR	1229	27% ≤ 19 years, 55% = 20–44 years, 18% ≥ 45 years.	455/774	Self‐report	Asthma = 89%, AR = 7%,	To determine the prevalence and common triggers of AR in the UAE	‐ Dust and grass/pollen were identified as common triggers for allergic rhinitis. ‐ Symptoms were most commonly reported during winter and spring.
Qasem et al. 2008^3^	Kuwait	Retrospective cross‐sectional	Asthmatic patients	4353	NS	NS	Collection of hourly spore and pollen counts, along with daily asthma visits, analyzed monthly in correlation with meteorological factors	‐ High pollen counts occurred in September, associated with a high number of patient visits. ‐ Fungal spore counts peaked in December, correlating with increased asthma‐related visits.	To evaluate the relationship between meteorological factors, aeroallergen levels, and asthma‐related healthcare visits in Kuwait	The study found that weather and aeroallergen levels significantly influence asthma‐related healthcare visits, with notable peaks in pollen and fungal spores correlating with increased patient visits.
Sharif et al. 2018^34^	UAE	Cross‐sectional	Children and adolescents with AR, asthma, or AD	180	1–16 years	73/107	SPTs	AR = 69.4%, Asthma = 52.2%, AD = 21.1%	To determine the most common aeroallergens causing sensitization in children in the UAE.	Sensitization to HDM was highest, followed by cat and dog allergens, with variations observed across different age groups.
Suliaman et al. 1996^35^	KSA	Cross‐sectional	Atopic population	1159	>5 years old	NS	SPTs	Saudi Arabs: *Chenopodium album* (53%), Kochia (51%), mesquite (46%), Cottonwood (38%), Alfalfa (36%), dust mite (Dermatophagoides farinae) (36%), cockroach (35%), house dust (31%), Bermuda grass (29%), Acacia (29%). Western Expatriates: Dust mite (Dermatophagoides farinae) (43%), house dust (41%), Alternaria (36%), Grass mix (34%), Bermuda grass (33%), mesquite (32%), Cat (31%).	To determine the pattern of immediate type hypersensitivity reactions among atopic populations in the Eastern province of Saudi Arabia, including comparisons between saudi Arabs and Western expatriates.	‐ Pollen hypersensitivity was prevalent among saudi Arabs, with high reactions to Chenopodium album and Kochia. Western expatriates showed significant hypersensitivity to dust mites, house dust, and molds like Alternaria. ‐ Mold and cat allergies were more common among Western expatriates than saudi Arabs, possibly due to environmental and lifestyle differences, such as the use of central air conditioning and the presence of domestic cats.
Tabbara et al. 2012^36^	Bahrain	Cross‐sectional	Children with asthma	95	6.8 ± 3.8	34/61	‐ Serum total and allergen‐specific IgE ‐ Eosinophilic count	Mild asthma = 71.6%, moderate asthma = 20.0%, severe asthma = 8.4%.	To profile the atopic conditions in asthmatic children and assess the prevalence of sensitization to aeroallergens and food allergens.	‐ High prevalence of sensitization to aeroallergens and food allergens among asthmatic children. ‐ A significant portion of the study population had mild asthma, and there was a notable association between asthma severity and age. ‐ Atopy and food allergies were common, with a significant family history of atopy noted in most cases.
Zahraldin et al. 2021^13^	Qatar	Retrospective chart review	Children with asthma and AR	473	7.6 ± 3.3	146/327	SPTs	Asthma = 37%, AR = 12%, Asthma and AR = 2%, Others = 0.8%.	To determine the patterns of aeroallergen sensitization and their relationship to clinical parameters in children with asthma and AR.	‐ Sensitization to house dust mite and American cockroach was more common in children aged ≤5 ‐ Sensitization to cat and dog allergens was more prevalent in children aged >10 years.

*Note*: Age presented as mean, mean (range), mean ± standard deviation, median (range) or as presented in the original study.

Abbreviations: AD, Atopic Dermatitis; AR, Allergic Rhinitis; CR, Chronic Rhinitis; HDM, House Dust Mite; IgE, Immunoglobulin E; ISAAC, International Study of Asthma and Allergies in Childhood; NS, Not specified; PB, Population Based; RAST, Radioallergosorbent Test; SPT, Skin Prick Test.

### 3.2 Quality of the studies and risk of bias

The majority of studies (23/27) were rated as high quality, suggesting minimal or no susceptibility to bias. None of the included studies were categorized as low quality or showed a high risk of bias. All studies met or exceeded the quality criteria outlined in the methods section (Table 2).

**TABLE 2:** Joanna Briggs Institute Critical Appraisal Checklist for Systematic Reviews (and risk of bias assessment).

	Q1	Q2	Q3	Q4	Q5	Q6	Q7	Q8		Risk of bias?	Quality
Quality appraisal table for cross‐sectional studies											
Al‐Dowaisan et al. 2004^14^	Y	Y	U	Y	N	N	Y	Y		Moderate (exposure)	Medium
Al‐Frayh et al. 1992^15^	Y	Y	Y	Y	Y	U	Y	Y		Low (selection of participants)	High
Al‐Frayh et al. 1999^16^	Y	Y	Y	Y	N	N	Y	Y		None	High
Al‐Ghamdi et al. 2022^17^	Y	Y	Y	Y	N	U	Y	Y		Moderate (random sampling)	High
AlKhater, 2017^18^	Y	Y	Y	Y	N	N	Y	Y		Low (demographic)	High
Almehizia et al. 2019^19^	Y	Y	Y	Y	Y	Y	Y	Y		Low (exposure)	High
Almogren, 2009^20^	Y	Y	Y	Y	N	N	Y	Y		None	High
Al‐Nesf et al. 2020^21^	Y	Y	Y	Y	N	N	Y	Y		Moderate (random sampling)	High
Al‐Nesf et al. 2022^22^	Y	Y	Y	Y	N	N	Y	Y		None	High
Alqahtani, 2016^23^	Y	Y	Y	Y	Y	Y	Y	Y		Low (data analysis techniques)	High
AlShatti and Ziyab, 2020^24^	Y	Y	Y	Y	Y	Y	Y	Y		None	High
Al‐Tamemi et al. 2008^26^, ^27^	Y	Y	Y	Y	N	N	Y	Y		Moderate (selection of participants)	Medium
AlMehdi et al. 2005^27^	N	Y	Y	Y	N	N	Y	Y		Low (selection of participants)	Medium
Hasan and Rizwan, 2015^12^	Y	Y	Y	Y	Y	Y	Y	N		None	High
Hasnain et al. 1994^28^	Y	Y	Y	Y	N	N	Y	Y		None	High
Hasnain et al. 1998^29^	Y	Y	Y	Y	N	N	Y	Y		Low (data analysis techniques)	High
Hasnain et al. 2004^30^	Y	Y	Y	Y	N	N	Y	Y		None	High
Hasnain et al. 2012^4^	Y	Y	Y	Y	N	N	Y	U		Low (data analysis techniques)	High
Kerkadi et al. 2009^31^	Y	Y	Y	Y	U	N	Y	Y		Moderate (random sampling)	High
Koshak, 2006^32^	Y	Y	Y	Y	Y	N	Y	N		Low (selection)	High
Mahboub et al. 2014^33^	Y	Y	Y	Y	N	U	Y	Y		Moderate (random sampling)	High
Qasem et al. 2008^3^	Y	Y	N	Y	N	N	Y	Y		Low (demographic, measurement)	Medium
Sharif et al. 2018^34^	Y	Y	Y	Y	N	N	Y	Y		Low (measurement)	High
Suliaman et al. 1996^35^	Y	Y	Y	Y	N	N	Y	U		None	High
Tabbara et al. 2012^36^	Y	Y	Y	Y	N	N	Y	Y		Low (follow up)	High
Zahraldin et al. 2021^13^	Y	Y	Y	Y	Y	Y	Y	Y		Low (demographic)	High

JBI critical appraisal checklist for Case control studies

Q1. Were the criteria for inclusion in the sample clearly defined?

Q2. Were the study subjects and the setting described in detail?

Q3. Was the exposure measured in a valid and reliable way?

Q4. Were objective, standard criteria used for measurement of the condition?

Q5. Were confounding factors identified?

Q6. Were strategies to deal with confounding factors stated?

Q7. Were the outcomes measured in a valid and reliable way?

Q8. Was appropriate statistical analysis used?

Q1. Were the groups comparable other than the presence of disease in cases or the absence of disease in controls?

Q2. Were cases and controls matched appropriately?

Q3. Were the same criteria used for identification of cases and controls?

Q4. Was exposure measured in a standard, valid, and reliable way?

Q5. Was exposure measured in the same way for cases and controls?

Q6. Were confounding factors identified?

Q7. Were strategies to deal with confounding factors stated?

Q8. Were outcomes assessed in a standard, valid, and reliable way for cases and controls?

Q9. Was the exposure period of interest long enough to be meaningful?

Q10. Was appropriate statistical analysis used?

**Table:** 

	Q1	Q2	Q3	Q4	Q5	Q6	Q7	Q8	Q9	Q10		Risk of bias?	Quality
JBI critical appraisal checklist for Case control studies													
Al‐Saleh et al. 2019^25^	Y	Y	Y	Y	Y	Y	Y	Y	Y	Y		Low (selection of participants)	High

### 3.3 Aeroallergen sensitivity

Table 1 presents the data extracted from the included studies, along with the key findings for each study.

**FIGURE 2:**
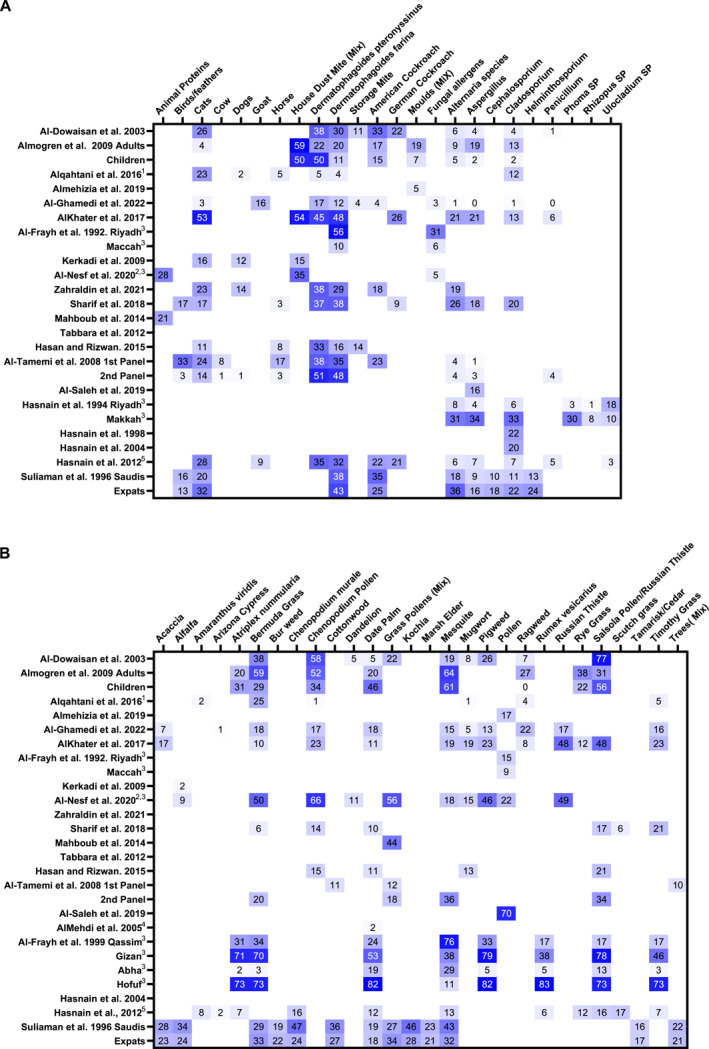
Distribution of reported results across study's included in the systematic review. (A) Reactivity to airborne animal, dust and mite allergens. (B) Reactivity to airborne plant allergens. %’s of sIgE positive subjects are shown following testing of sIgE serum levels, except where otherwise indicated. Heatmap plots data from where more than two results for the same allergen were reported. ^1^Calculated the total % for combined A.D.s, ^2^% within pollen sensitised group, ^3^SkinPrick Testing was used, ^4^% RAST POSITIVE of Airborne Allergen Allergic, ^5^Study used reported averages from multiple sites.

Aeroallergen sensitization was primarily assessed via skin prick testing (SPT) in 15 studies,^4^, ^12^, ^13^, ^14^, ^15^, ^18^, ^20^, ^21^, ^23^, ^26^, ^28^, ^29^, ^30^, ^34^, ^35^ with 5 using in vitro methods,^17^, ^25^, ^27^, ^31^, ^36^ 2 employing both approaches,^16^, ^32^ 4 relying on self‐reports^19^, ^22^, ^24^, ^33^ One study conducted aerobiological monitoring with meteorological analysis to characterize allergen presence rather than sensitization.^3^ Sensitization rates varied considerably, influenced by factors such as age, demographics, and location. House dust mite sensitization ranged from 14.8% to 77.8%, depending on the region and population. Up to 65.1% of allergen‐positive individuals demonstrated polysensitization. Sensitization patterns differed between indigenous populations and expatriates, with local allergens being more prevalent among natives. Sensitization rates were lower in younger children but increased with age. Detailed sensitization rates per allergen are provided in Figure 2.

## 4 DISCUSSION

### 4.1 Testing methodology for aeroallergen sensitization

Methods used included detection of specific IgE antibodies through skin prick testing (SPT) with commercial or in‐house extracts, in vitro testing, or through self‐reported symptoms by subjects. In vitro testing was performed by enzyme immunoassay or radioallergosorbent assay (Table 1). It is generally considered that for aeroallergens, in vitro methods perform at a similar high level to SPT, though there can be different sensitivity/specificity for individual allergens.^37^, ^38^ It is not possible to draw conclusions about comparability in this review, though Koshak (2006)^32^ reported a significant correlation between positive SPT and the Phadiatop in vitro allergen screening panel. Self‐reporting was used in 4 studies,^19^, ^22^, ^24^, ^33^ and we consider that methodology, while useful in some ways, does not provide a truly comparable picture of allergic sensitization as detection of specific IgE antibodies.

This review addressed‐allergic sensitization, rather than clinical allergy. As expected, the rates of the former will be relatively higher for example, sensitization was detected in 223 of the 505 subjects (44.2%) screened in a study population of blood donors in Kuwait. However, the prevalence of current or previous‐allergic disease (subject‐reported) was much lower at 20.6%.^39^

### 4.2 Prevalence of aeroallergen sensitization

Because the range of tested allergens differed between the studies, it is not possible to extract a range of aeroallergen sensitization for all allergens. However, estimates can be extracted for the more common allergens (Figure 2). There is a wide range in the prevalence of aeroallergen sensitization between the studies. Multiple factors may contribute to this variability, including the age range and demographic composition, the size of the study population, the distribution of atopic conditions within the cohort, the geographical location of the study itself, and the degree of urbanization. For example, house dust mite sensitization varied from 14.8% in a pediatric study in Najran in Southwestern KSA to 77.8% in a combined adult and adolescent study in Riyadh, central KSA.^20^, ^23^ Within an adult multi‐center study, mite sensitization varied from 30% in Jeddah, 46% in Dubai, and 72% in Khartoum.^4^ It is very clear that there is substantial variation picture within the GCC, which should not be unexpected given the variation in climate, geography, and rural versus urban populations.^15^, ^16^

It is interesting to consider whether in patients from the GCC in a desert climate, there would be similarities in allergen sensitization to Western populations. Indeed, several studies noted the high prevalence of sensitization to certain common allergens such as house dust mite, cat dander, aspergillus and Cladosporium. It has been noted that some Western plants were deliberately imported into the region in order to “green” the desert, which may contribute to sensitization.^14^ Multiple studies assessed allergens local to the region, which would not be assessed in Western studies. Locally relevant allergens with high sensitization prevalence include Chenopodium, Salsola, Prosopis, and Phoenix dactylifera species.^4^, ^16^, ^26^, ^35^, ^40^

Studies assessing asthmatic patients alone should not be directly compared to studies including all atopic conditions, as sensitization rates will vary in patients with allergic rhinitis, or eczema, or in patients with multiple atopic conditions. There were several asthmatic studies focused purely on sensitization to fungi, or even more focused on particular fungal species.^4^, ^25^

Several studies commented on the presence of polysensitization. For example, Ezeamuzie et al. (2000)^40^ reported that in 109 allergen‐positive subjects, 71 (65.1%) were sensitized to more than one allergen, of which 30 (27.5%) were sensitized to ≥ 4 allergens. In a study of 473 children in Qatar with asthma and AR, Zahraldin et al. (2021)^13^ noted 204 (43.1%) to be monosensitized, 215 (45.5%) sensitized to 2‐3 allergens, and 54 (11.4%) sensitized to ≥ 4 allergens.

### 4.3 Study population demographics

An interesting concept is to consider the relative contribution to the data in those studies which did distinguish between the indigenous population and expatriates. The different genetic background and previous allergenic exposure may differ substantially between such groups. Ezeamuzie et al. (1997)^39^ reported a higher prevalence of aeroallergen sensitization (50.2%) in Kuwaiti nationals compared with non‐Kuwaitis (34.2%) (chi^2^ = 8.6, *p* < 0.003). Sensitization increased with age only among the expatriate study population. Suliaman et al. (1997)^35^ assessed 806 Saudi Arabs and 241 western expatriates, mostly from North America. The 10 most common allergens differed substantially between populations, with local allergens (Chenopodium, kochia, mesquite) dominating in natives, while dust mite‐farinae, dust mite, alternaria, and cat predominated in the expatriates. Such differences are important for clinical assessment and testing. Furthermore, depending on the specific GCC country, the proportion of expatriates differs widely. Thus, this may have implications for the volume of laboratory testing for different allergens, the use of different allergen panels, and ordering of different skin test reagents. Customized panels may be both more clinically relevant and more economically sound.

The age of the patient may well impact sensitization prevalence; in our review, there were 7 adult studies, 10 pediatric, and 10 including both adults and children. Younger children may not have had sufficient exposure to develop allergic responses. For example, Kerkadi et al.^31^ reported sensitization prevalence in of 14.9% for dust in a Qatari pediatric study (mean age 4.5+/−0.3 years), whereas a Qatari study of older children (age 7.6+/−3.3 years) showed 38.1% to dust mites, and an adult study from Qatar noted a prevalence of 34.9%.^13^, ^21^ However, other points to consider are that the proportion of allergic rhinitis patients was higher in the latter 2 studies, and that these 2 studies were reported more than 10 years later, which might reflect changing patterns in urbanization, pollution, and general atopic sensitization.

Sex differences in allergic rhinitis have been reported in two studies, highlighting distinct patterns in triggers, severity, and symptom reporting. Almehizia et al. 2019^19^ found that males were more likely to have pollen as a trigger (20.6%) compared to females (14.1%, *p* < 0.001), while females reported dust as a trigger more frequently (77.8% vs. 69.3%, *p* < 0.001). Regarding severity, males were more prone to moderate to severe allergic rhinitis (70.7%), whereas females were less likely to report severe symptoms (adjusted odds ratio 0.8, *p* = 0.006), suggesting that males experience more intense forms of allergic rhinitis, particularly when triggered by pollen. The study by Alqahtani 2016,^23^ on 1700 students, found that boys were more likely to have diagnosed allergic rhinitis (*p* = 0.001) and reported higher rates of wheezing, exercise‐induced wheeze, and night cough compared with girls.

Both studies demonstrate that males tend to have more severe allergic rhinitis and experience more intense respiratory symptoms, whereas females are more likely to report dust as a trigger and experience less severe symptoms. These findings highlight the importance of considering sex in allergic rhinitis diagnosis and management for more personalized care.

### 4.4 Strength and limitations

This systematic review is robust because it uses a thorough approach, with a solid search strategy across multiple databases and follows PRISMA guidelines. It is also the first to address aeroallergen sensitization in the GCC region. By examining both native and expatriate populations, it provides important insights into differences in aeroallergen sensitization, which is essential for improving clinical care.

However, there are limitations. Most studies were cross‐sectional, but only two were chart reviews and one was a case‐control study, which might affect how results compare. Also, some studies relied on self‐reported data, which can lead to bias. Although all studies were rated as high or medium quality, limited data on aeroallergens in some GCC countries may reduce the overall conclusions. Future research should fill these gaps and further investigate aeroallergen sensitization in this region.

## 5 CONCLUSION

The importance of this systematic review can be considered in various aspects, by providing locally relevant allergen sensitivity, which can aid clinicians and scientists in the following ways:

- To improve clinical diagnosis via clinically relevant testing to a specific city, region, or country.
- To be aware of the potential differences in sensitization between native and expatriate patients.
- To make potential cost savings by undertaking appropriate allergen testing, without testing for allergens which are not relevant.
- To consider customised panels based on geographical location, age, and native/expatriate status, which may be both more clinically relevant and more economically sound.
- To provide appropriate advice regarding allergen avoidance.
- To optimise the selection of allergens for specific immunotherapy.
- To apply pressure on manufacturers of skin prick tests and specific IgE allergen reagents to produce high‐quality products relevant to the GCC population.

Our systematic review highlights the crucial importance of providing allergen‐sensitivity information that is specifically tailored to the local environment. This tailored approach can have numerous advantages, including improved clinical diagnosis by conducting precise testing that is relevant to the specific city, region, or country. Allowing for the recognition of potential variations in sensitization between native individuals and expatriates and resulting in potential cost savings by ensuring that only relevant allergens are tested, thus avoiding unnecessary testing for irrelevant allergens. Furthermore, considering customized panels based on geographical location, age, and native/expatriate status can not only offer more clinically relevant results but also prove to be economically sound. Additionally, providing appropriate advice regarding allergen avoidance and optimizing the selection of allergens for specific immunotherapy can significantly enhance patient care. Lastly, by advocating for the production of high‐quality allergen testing products relevant to the GCC population, the review identifies a potentially significant way in which to improve healthcare outcomes in the region.

## AUTHOR CONTRIBUTIONS

**Tahira Khurram**: Methodology; writing—original draft; data curation; investigation; formal analysis. **Ghalia Missous**: Methodology; data curation; writing—original draft; investigation; formal analysis. **Nicholas van Panhuys**: Methodology; writing—review and editing; supervision; formal analysis; data curation. **Mohammed Yousuf Karim**: Methodology; supervision; writing—original draft; writing—review and editing; formal analysis.

## CONFLICT OF INTEREST STATEMENT

The authors declare no conflicts of interest.

## KEY MESSAGE

Aeroallergen sensitization is high in the GCC, with dust mites as the primary trigger; regional studies emphasize the need for standardized diagnostics and region‐specific management to address climate and lifestyle influences.

## Data Availability

Data sharing is not applicable to this article as no new data were generated.
